# Evolutionary constraint and population variation across coagulation genes

**DOI:** 10.1016/j.rpth.2026.106980

**Published:** 2026-09-24

**Authors:** Omid Seidizadeh, Himani Chand, Andrea Cairo, Flora Peyvandi

**Affiliations:** 1 Angelo Bianchi Bonomi Hemophilia and Thrombosis Center, Fondazione IRCCS Ca’Granda Ospedale Maggiore Policlinico, Milan, Italy; 2 Department of Pathophysiology and Transplantation, Università degli Studi di Milano, Milan, Italy

**Keywords:** coagulation genes, genomics, gnomAD constraint, hemostasis, molecular evolution

## Abstract

**Background:**

Hemostasis is a tightly regulated system in which disruption of coagulation genes can cause bleeding or thrombotic disorders. Despite extensive variant discovery, it remains unclear whether coagulation genes share uniform or gene-specific patterns of evolutionary constraint and population variation.

**Objectives:**

To characterize evolutionary constraint and population variation across coagulation genes using large-scale genomic data.

**Methods:**

We performed a population-genomic analysis of 34 genes associated with inherited bleeding, fibrinolytic, and thrombotic disorders using Genome Aggregation Database (gnomAD) v4.1.1 data from more than 800,000 individuals. We evaluated gene-level constraint metrics, including loss-of-function observed/expected ratios, loss-of-function observed/expected upper bound fraction, missense constraint scores, and synonymous variation metrics. We quantified rare and very rare variant burden after normalization for coding sequence length, assessed predicted functional impact using CADD and REVEL scores, evaluated nucleotide-level evolutionary conservation using phylogenetic *P* value scores, and characterized ClinVar pathogenic, uncertain, and benign variants identified in gnomAD.

**Results:**

Hemostatic genes demonstrated a continuous spectrum of evolutionary constraint rather than uniform intolerance to genetic variation. Genes including *F8, F9, F2, VWF, MYH9, SERPINC1,* and *PROS1* showed strong depletion of damaging variation, whereas *GP6, GP1BB, F12, VKORC1, MCFD2,* and *LMAN1* showed greater tolerance. Rare variant density differed substantially across genes even after adjustment for coding sequence length. ClinVar pathogenic variants identified in gnomAD were enriched at evolutionarily conserved sites and displayed marked gene-specific differences in variant class distribution, particularly among missense variants.

**Conclusions:**

The hemostatic system is shaped by heterogeneous evolutionary pressures, resulting in gene-specific patterns of constraint and variation tolerance. Integrating population genomics, evolutionary conservation, and clinical annotations provides a framework for understanding genetic variation across bleeding, thrombotic, and fibrinolytic disorders.

## Essentials

• It is unclear whether coagulation genes share similar patterns of constraint and variation.
• We analyzed population variation in 34 hemostatic genes in over 800,000 individuals.
• Hemostatic genes showed marked differences in tolerance to damaging variants.
• Pathogenic variants were enriched at evolutionarily conserved sites across genes.

## 1 Introduction

Hemostasis is a tightly regulated system that preserves vascular integrity while preventing pathologic thrombosis. It is governed by a network of coagulation factors, platelet receptors, anticoagulant pathways, and fibrinolytic components that maintain the balance between bleeding and clot formation [1]. Disruption of this balance leads to a spectrum of disorders, ranging from severe bleeding diseases caused by variants in genes such as *F2*, *F8*, *F9*, and *VWF*, to thrombotic conditions associated with genes including *SERPINC1*, *PROC*, and *PROS1* [[2], [3], [4]]. Most inherited bleeding disorders follow recessive or X-linked inheritance patterns, although several disorders, including von Willebrand disease and platelet disorders, show dominant inheritance. Conversely, many inherited thrombophilic disorders display autosomal dominant inheritance with variable penetrance. Although many disease-associated variants have been identified over the past decades, it remains unclear whether coagulation genes share uniform or gene-specific patterns of evolutionary constraint and population variation, and how this heterogeneity shapes the interpretation of genetic variants in bleeding and thrombotic disorders.

Large-scale human sequencing datasets now provide unprecedented opportunities to study the genetic landscape of disease-associated genes at population scale. Among these resources, the Genome Aggregation Database (gnomAD) aggregates exome/genome sequencing data from more than 800,000 individuals and offers a powerful framework to investigate mutational constraint, variant frequency spectra, and selective pressures across the human genome [[5], [6], [7]]. Constraint metrics derived from such datasets provide insights into the tolerance of genes to different classes of variants, including predicted loss-of-function (LoF), missense, and synonymous variants [[5], [6], [7]]. Genes that exhibit strong depletion of functional variants are typically under purifying selection and are often enriched for essential biological functions and Mendelian disease associations.

While constraint-based analyses have been widely applied across the genome [[5], [6], [7]], systematic investigation of coagulation genes in this context remains limited. The hemostatic system is particularly interesting from an evolutionary perspective because it is shaped by competing selective forces: variants that impair coagulation predispose to bleeding disorders, whereas hypercoagulable states increase thrombotic risk. This evolutionary tension may have shaped the genetic architecture of coagulation genes, leading to distinct patterns of constraint and variability across the hemostatic network and informing the interpretation of variants identified in clinical sequencing.

Beyond gene-level constraint, integrating population and evolutionary data provides deeper insight into the functional landscape of coagulation genes. Population frequency data can reveal rare variants in apparently healthy individuals, including those previously classified as pathogenic or likely pathogenic in ClinVar, helping refine disease prevalence estimates and variant interpretation [[8], [9], [10], [11], [12], [13]]. Computational predictors such as Combined Annotation-Dependent Depletion (CADD) and Rare Exome Variant Ensemble Learner (REVEL) scores further assess the potential functional impact of rare variants [14,15]. In addition, evolutionary conservation metrics such as phylogenetic *P* value (PhyloP) provide nucleotide-level evidence of selective constraint across species, highlighting genomic positions under strong purifying selection [16]. Integrating population-level variants, mutational constraint, clinical pathogenicity annotations, computational predictions, and evolutionary conservation provides a powerful framework to dissect the genetic architecture of disease-associated genes.

In this study, we performed a comprehensive population-genomic and evolutionary analysis of genes involved in inherited bleeding, fibrinolytic, and thrombotic disorders. Using data from gnomAD v4.1.1, we evaluated gene-level constraint metrics for 34 genes, assessed the presence and allele frequencies of variants reported as pathogenic or likely pathogenic in ClinVar, and characterized the spectrum of rare variants across coagulation-related genes. We further examined the predicted functional impact of these variants using the CADD score and REVEL score and investigated nucleotide-level evolutionary constraint using PhyloP. By integrating these complementary genomic and evolutionary layers, we aimed to provide a comprehensive view of the evolutionary landscape and genetic architecture of coagulation genes.

## 2 Methods

### 2.1 Selection of coagulation gene list and data analyses

We selected a comprehensive set of coagulation-related genes using the International Society on Thrombosis and Haemostasis Tier 1 gene list (https://www.isth.org/page/GinTh_GeneLists), which comprises genes with strong clinical evidence in bleeding, fibrinolysis, and thrombotic disorders. From this resource, we selected well-characterized genes with established roles in inherited hemostatic phenotypes, prioritizing those supported by robust clinical and functional data while excluding genes with limited or indirect evidence. In total, 34 genes were included, representing key coagulation factors, fibrinolysis, platelet receptors, and anticoagulant pathways (Supplementary Table).

All analyses were performed using the GRCh38/hg38 genome build. gnomAD v4.1.1 data were accessed via the public web interface (https://gnomad.broadinstitute.org/), and gene-specific variant datasets were downloaded individually in CSV format for each gene. Transcript selection was based on the International Society on Thrombosis and Haemostasis Tier 1 gene list and corresponding canonical disease-relevant transcripts as defined in the gold-standard gene set, with full transcript information provided in the Supplementary Materials. Variant filtering criteria were applied based on global allele frequency thresholds, defining rare variants as those with allele frequency <0.01 and very rare variants as those with allele frequency <0.0001. Functional annotation was performed using Variant Effect Predictor. Throughout the manuscript, LoF variants refer to high-confidence predicted LoF variants as defined in gnomAD v4.1.1. The gnomAD constraint and population-level metrics were used as provided in the dataset without additional transformation or adjustment. Computational analyses and visualization were performed in R version 4.5.1 (2025-06-13) using RStudio 2026.05.1+225 “Golden Wattle” Release. Key packages included biomaRt (v2.66.2) for gene and transcript annotation and ggplot2 (v4.0.3) for data visualization (Supplementary Figure S1).

### 2.2 Analysis of gene-level constraint metrics from gnomAD

To evaluate gene tolerance to different classes of genetic variation (ie, LoF, missense, and synonymous), we obtained gene-level constraint metrics from gnomAD v4.1.1. For genes lacking available metrics in gnomAD v4.1.1, we used corresponding metrics from gnomAD v2.1.1 (ie, LoF constraint metrics for *GP9*). These metrics provide indirect evidence of selective constraint by comparing observed variant counts with those expected under a neutral mutational model [[5], [6], [7]]. This approach allows the identification of genes under strong purifying selection as well as those more permissive to genetic variation. We retrieved the following metrics for each gene:

(1) LoF observed/expected (LoF o/e) ratio—quantifies depletion of predicted LoF variants.
(2) Loss-of-function observed/expected upper-bound fraction (LOEUF)—the upper bound of the 90% confidence interval of the LoF o/e ratio, widely used to classify genes by intolerance to LoF variants.
(3) Missense or synonymous observed/expected (o/e) ratio—quantifies depletion of missense or synonymous variants.
(4) Missense or synonymous Z scores, which capture the deviation between observed and expected variant counts, thereby reflecting the degree of selective constraint acting on coding and neutral variants.

### 2.3 Quantification of rare and very rare variant burden in gnomAD across coagulation genes

We assessed the burden of rare (minor allele frequency [MAF] < 0.01) and very rare (MAF < 0.0001) variants across these 34 genes to characterize patterns of genetic variation in coagulation-related loci in more than 800,000 individuals in the gnomAD population. This analysis allows us to identify genes that are more tolerant to (very) rare variation vs those under stronger selective constraint, providing insight into their relative susceptibility to deleterious variants and potential contribution to bleeding, fibrinolysis, or thrombotic phenotypes. These analyses were adjusted for the coding sequence (CDS) length of each gene.

### 2.4 Computational assessment of variant pathogenicity and evolutionary constraint

We conducted computational annotation of all rare and very rare variants in the selected coagulation genes, applying REVEL and CADD scores to missense variants and PhyloP across all classes of coding and noncoding variants [[15], [16], [17]]. REVEL and CADD provide quantitative predictions of the potential deleteriousness of variants, with higher scores (REVEL > 0.5, CADD > 20) indicating greater likelihood of functional impact. We hypothesized that genes under stronger constraint would show greater depletion of variants predicted to be deleterious, as assessed by REVEL and CADD scores, in the gnomAD dataset.

PhyloP scores were used to quantify evolutionary conservation or acceleration at individual nucleotide positions across multiple species [16]. PhyloP is a likelihood ratio–based method that evaluates each nucleotide site by comparing the probability of the observed substitution pattern under a neutral evolutionary model vs a model that allows either conservation or acceleration, using a phylogenetic tree of multiple species. The scores correspond to -log_10_(*P* value) under a neutral model, such that negative scores indicate accelerated evolution, suggesting reduced functional constraint and a higher tolerance to variation. Scores close to zero are consistent with neutral evolution, whereas positive scores reflect evolutionary constraint, indicating that variants in these regions are subject to purifying selection and are more likely to have functional consequences. In particular, phyloP scores ≥2.27 (∼*P* ≤ .005) are considered to represent sites under significant evolutionary constraint [17].

Based on this framework, we evaluated the distribution of phyloP scores among all types of variants observed in the gnomAD general population with MAF < 0.01 (rare) or < 0.0001 (very rare) to explore patterns of evolutionary constraint across genes. We hypothesized that genes under strong selective constraint show a relative depletion of variants at highly conserved sites, resulting in a shift of observed variants toward lower phyloP scores. However, given that rare and ultrarare variants represent recent mutational events, variants at conserved positions may still be observed prior to complete purifying selection. Accordingly, the distribution of phyloP scores in gnomAD reflects the combined effects of mutational processes and ongoing selection, rather than a strong measure of gene-level constraint alone.

### 2.5 Assessment of ClinVar variant representation in gnomAD

We systematically examined the presence of pathogenic/likely pathogenic (from now on, pathogenic) variants reported in ClinVar that have a MAF < 0.01 or < 0.0001 in gnomAD for our curated list of coagulation genes. This analysis aimed to assess how selective constraint and evolutionary conservation shape the distribution of clinically relevant variants in the general population. We hypothesized that highly constrained and evolutionarily conserved genes would harbor fewer pathogenic variants in gnomAD, as deleterious mutations in these genes are less likely to be tolerated. To test this, we calculated the following, for each gene: (1) the total number of pathogenic variants reported in ClinVar, (2) the total number of benign or likely benign variants for comparison, and (3) the ratio of pathogenic to benign variants. We further integrated these counts with gene-specific metrics such as CDS length to control for size-related effects, enabling fair comparisons across genes. This approach allowed us to investigate whether genes under stronger constraint and higher evolutionary conservation indeed carry fewer clinically significant variants in the general population, and to explore patterns of variant distribution and variant types that may inform disease gene interpretation and clinical variant classification.

This study used publicly available, aggregated genomic data from the gnomAD database and did not involve direct recruitment of participants or access to identifiable individual-level data. Therefore, ethics committee approval and informed consent were not required for this study.

## 3 Results

### 3.1 Selection of coagulation gene list

We selected 34 genes causing either inherited bleeding (*F2, F5, F7, F8, F9, F10, F11, F12, F13A1, F13B, FGA, FGB, FGG, GGCX, VKORC1, GP1BA, GP1BB, GP6, GP9, ITGB3, ITGA2B, MYH9, LMAN1, MCFD2, VWF,* and *SERPINF2*) or inherited fibrinolytic/thrombotic complications (*ADAMTS13, MTHFR, PLG, PROC, PROS1, SERPINC1, SERPINE1,* and *PLAT)* for our analyses. Gene function, encoded proteins, associated disorders, and modes of inheritance are summarized in the Supplementary Table.

### 3.2 Constraint metrics reveal gene-specific tolerance to variation

#### 3.2.1 LoF variants (o/e ratio and LOEUF)

We assessed the observed/expected (o/e) ratio and LOEUF (the upper bound of the 90% confidence interval of the o/e ratio) for LoF variants to quantify a gene’s intolerance to LoF variation. Values <1 for the o/e ratio indicate depletion and thus selective constraint. Recommended thresholds for LOEUF are <0.6 for gnomAD v4 and <0.35 for v2. Lower LOEUF indicates strong constraint against LoF variants, whereas higher values reflect greater tolerance to LoF variants. In this study, several bleeding-related genes, including *F9, F8, MYH9, SERPINF2, FGG, F2, FGB*, and *LMAN1* displayed very low o/e and LOEUF values, consistent with strong purifying selection and high intolerance to LoF variants. Genes such as *VWF, ITGB3, F13A1,* *ITGA2B*, and *F5* exhibit intermediate LOEUF scores, reflecting moderate constraint. By contrast, *GP1BB, GP9,* *GP1BA,* *GP6, MCFD2, FGA, VKORC1, F11, F12, F10,* and *F7* had higher LOEUF values, indicating relative tolerance to LoF variation (Figure 1A).

**Figure 1:**
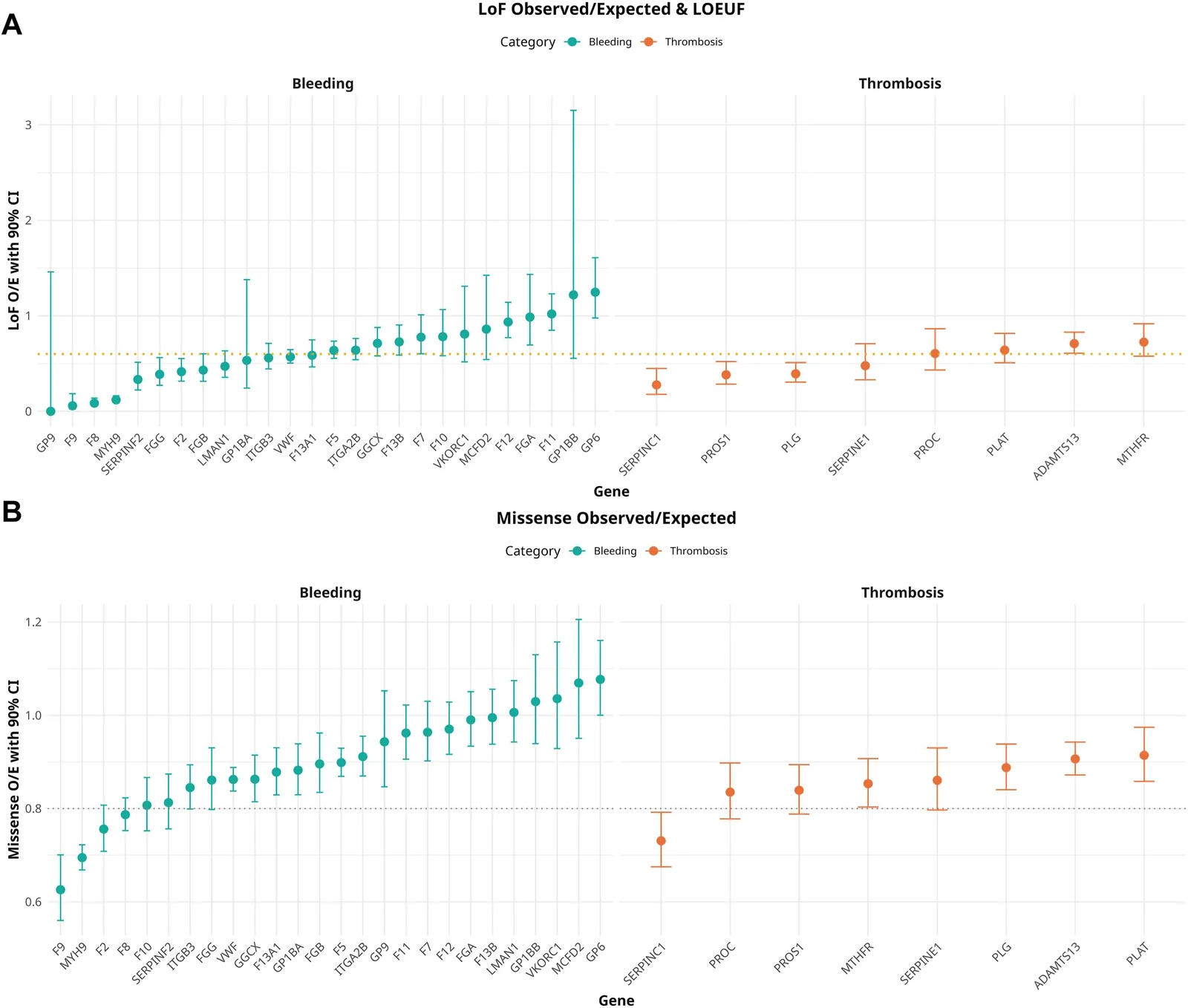
Gene-level constraint metrics for loss-of-function (LoF) variants (observed/expected ratio and loss-of-function observed/expected upper bound fraction [LOEUF]) and missense variants (observed/expected ratio). (A) LoF constraint reveals strong intolerance in several bleeding and thrombotic genes (eg, F9, *F8, MYH9, SERPINF2, FGG, F2,* and *FGB*), intermediate constraint in genes such as *LMAN1, ITGB3, VWF,* and *F5*, and relative tolerance in others (eg, *GP6, GPIBB, GP9, F11, F12,* and *VKORC1*), with confidence intervals reflecting variability in estimation. LOEUF represents the upper bound of the 90% confidence interval of the observed/expected (o/e) ratio for LoF variants. (B) Missense constraint shows a similar pattern, with substantial depletion in key genes (eg, *F9, MYH9, F2,* and *F8*), moderate constraint in many coagulation genes, and relative tolerance in a subset, while fibrinolysis-related genes generally exhibit consistent but variable missense constraint.

Notably, the 90% CIs highlight variability in constraint estimates. Wider CIs (eg, *GP9, GP1BB, GP6, FGA, GP1BA, MCFD2,* and *VKORC1*) likely reflect limited power for precise estimation, whereas narrower CIs (eg, *F9, F8, MYH9, VWF,* and *F5*) indicate more robust estimates driven by greater variant counts and gene coverage in gnomAD.

Among fibrinolysis/thrombotic genes, *SERPINC1, PROS1,* and *PLG* showed very low o/e and LOEUF values, consistent with strong purifying selection for LoF variants, whereas *SERPINE1* demonstrated intermediate constraint. Genes including *MTHFR, ADAMTS13, PLAT,* and *PROC* had higher values, indicating a slight tolerance to LoF variants (Figure 1A).

#### 3.2.3 Missense variants (o/e ratio and Z score)

We next assessed selective constraint on missense variants using the o/e ratio and Z score from gnomAD. The o/e ratio compares observed to expected missense variants under a neutral model; values <1 indicate depletion and thus selective constraint. Among bleeding-related genes, several, including *F9, MYH9, F2,* and *F8,* showed o/e ratios ≤0.8, indicating substantial depletion of missense variants. Many other genes exhibited o/e ratios between 0.8 and 1 (eg, *F10, SERPINF2, ITGB3, FGG, F13A1, VWF,* and *F5*), suggesting mild to moderate constraint. In contrast*, GP6, MCFD2, VKORC1, GP1BB,* and *LMAN1* had o/e ratios greater than 1, with *GP6* showing the most pronounced ratio (Figure 1B). Among fibrinolysis/thrombotic-related genes, all displayed o/e ratios below 1, indicating some degree of missense constraint across the group, although only *SERPINC1* showed a ratio below 0.8 (Figure 1B).

In addition to o/e ratios, gnomAD provides missense Z scores, which more precisely quantify the deviation of observed missense variation from expectation. Positive scores indicate intolerance, whereas negative scores suggest relative tolerance to missense variants; a Z score >3 is considered strong evidence of constraint. Consistent with the o/e results, several bleeding-related genes including *GP6, MCFD2, VKORC1, GP1BB,* and *LMAN1* displayed negative Z scores, indicating relative tolerance to missense variation. In contrast, most other bleeding-related genes showed positive Z scores, suggesting varying degrees of constraint. Notably, only *MYH9* (Z of 6.63), *F8* (Z of 3.77), *VWF* (Z of 3.55), and *F2* (Z of 3.01) exceeded the threshold for strong missense constraint, with some other genes close to this threshold, such as *F9, F5, F10,* and *ITGB3* (Figure 2A). Among fibrinolysis/thrombotic-related genes, all exhibited positive Z scores, again consistent with the o/e analysis. However, none reached the strong-constraint threshold of >3, with the highest value observed in *SERPINC1* (Z of 2.76, Figure 2A).

**Figure 2:**
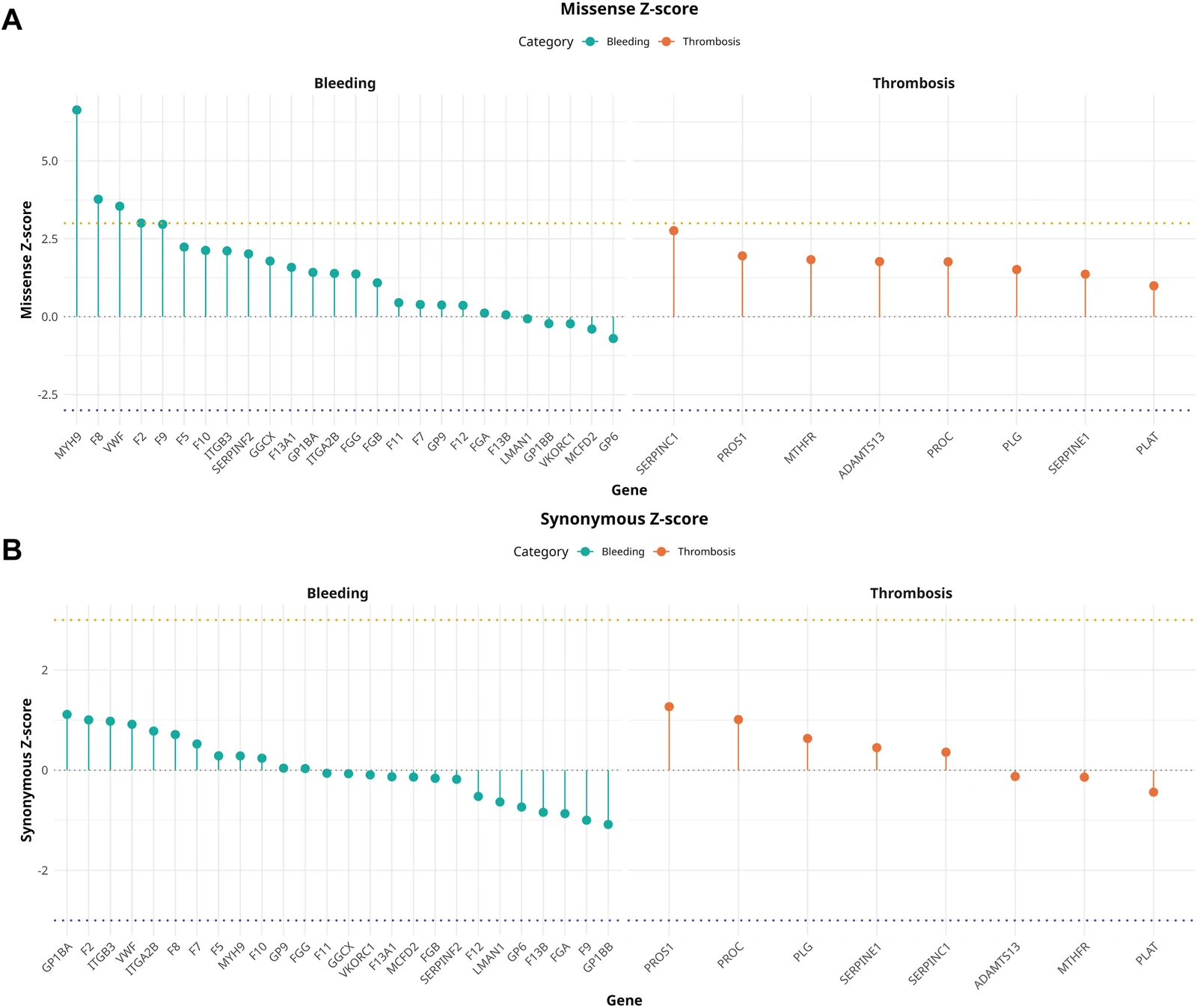
Z scores for missense and synonymous variants. (A) Missense Z scores indicate variable constraint across genes, with strong intolerance observed in *MYH9, F8, VWF,* and *F2* (Z > 3), moderate constraint in several bleeding-related genes (eg, *F9, F5,* and *F10*), and relative tolerance reflected by negative Z scores in genes such as *GP6, MCFD2, VKORC1,* and *GP1BB*; thrombosis and fibrinolysis-related genes show consistent but weaker constraint. (B) In contrast, synonymous Z scores are largely centered around zero, indicating minimal constraint, with only modest depletion or excess observed in a subset of genes.

#### 3.2.4 Synonymous variants (o/e ratio and Z scores)

We next evaluated selective constraint against synonymous variation using o/e ratios and synonymous Z scores. As expected under neutrality, most coagulation-related genes exhibited o/e ratios close to 1, indicating minimal deviation from expectation (Supplementary Figure S2). However, a subset of genes showed modest departures from neutrality: *F9, GP1BB*, *GP6*, *F13B*, *FGA,* and *F12* displayed an elevated o/e ratio, suggesting an excess of synonymous variation, while several genes, including *GP1BA, F2, ITGB3, ITGA2B, F7, F8, VWF,* and *F10,* showed slight depletion (o/e <1; Supplementary Figure S2). Among thrombotic/fibrinolysis genes, o/e ratios were generally tightly clustered around 1, with *PLAT, MTHFR*, and *ADAMTS13* showing a mild excess and *PROS1* the lowest value (Supplementary Figure S2).

Consistent with these findings, synonymous Z scores were centered around zero for most genes, indicating weak or absent constraint. A few genes, including *GP1BA, F2, ITGB3, VWF, ITGA2B, F8, F7,* and *F5*, showed modest positive Z scores, suggesting slight depletion of synonymous variants, whereas *GP1BB, F9, FGA, F13B, GP6* (and to some extent *LMAN1, F12, ADAMTS13, MTHFR,* and *PLAT*) exhibited negative Z scores, consistent with a slight excess of observed synonymous variants (Figure 2B).

### 3.3 Burden of rare and very rare variants across coagulation genes in gnomAD

The burden of all rare (MAF < 0.01, *n* = 92,678) and very rare (MAF < 0.0001, *n* = 90,533) variants across 34 coagulation genes in gnomAD was investigated (Figure 3). We found a marked variability in the number of both very rare and rare variants across the gene list, partly driven by gene size. The highest counts were observed in *VWF, MYH9, ADAMTS13, F5, ITGA2B*, and *F8* (Figure 3A, B*)*. After normalization for CDS length of each gene, several genes such as *F8, F9, GP1BA, GP9, F5*, *FGA,* and *SERPINC1* exhibited the lowest variant density, suggesting a stronger selective constraint for (very) rare variants. In contrast, *GP6, ITGA2B, PLAT, LMAN1, F12, MCFD2, ADAMTS13,* and *VKORC1* showed higher normalized burdens, indicating greater tolerance to (very) rare variation beyond the effect of gene size.

**Figure 3:**
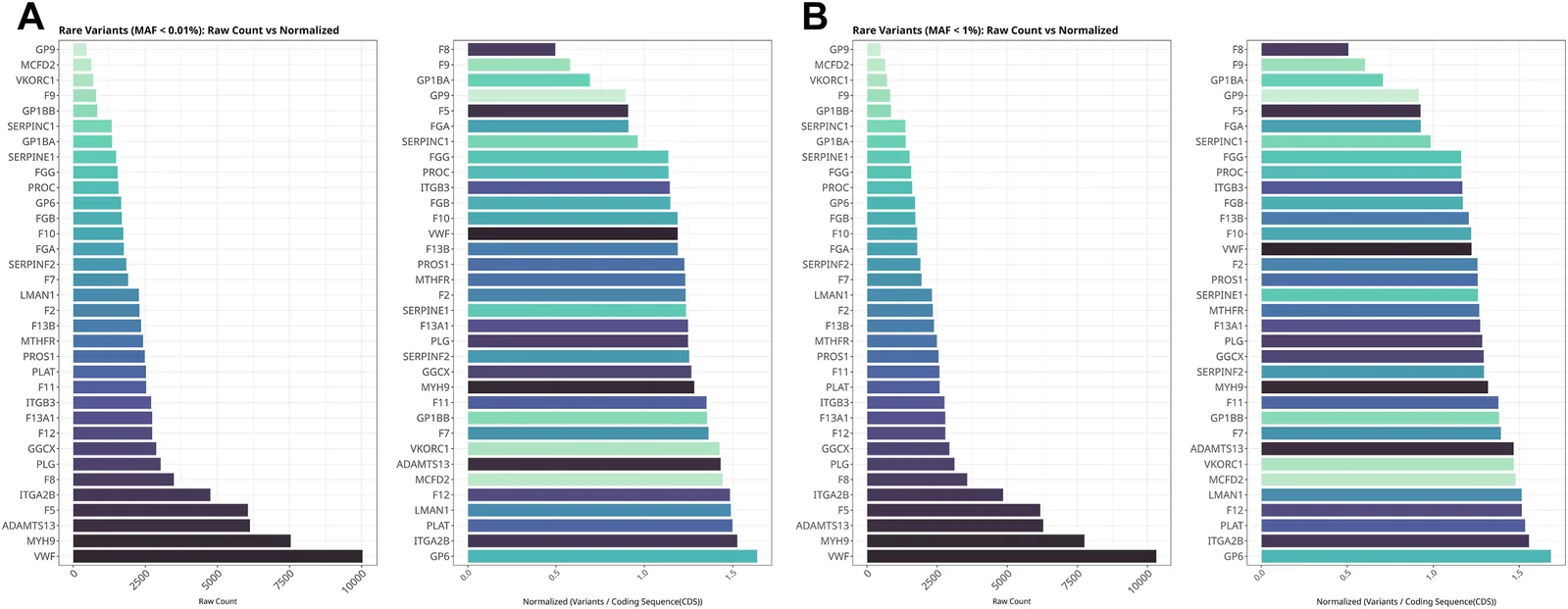
Burden of rare and very rare variants across coagulation genes in Genome Aggregation Database. The distribution of very rare (minor allele frequency [MAF] < 0.0001; A) and rare (MAF < 0.01; B) variants shows substantial variability across genes, largely influenced by gene size, with the highest counts observed in larger genes (eg, *VWF, MYH9, ADAMTS13, F5,* and *F8*). After normalization for coding sequence (CDS) length of each gene, several genes (eg, *F8, F9, GP1BA, GP9,* and *SERPINC1*) display lower variant density consistent with stronger constraint, whereas others (eg, *GP6, ITGA2B, LMAN1, F12,* and *VKORC1*) exhibit higher burdens, indicating relative tolerance to rare variation.

### 3.4 *In silico* prediction of pathogenicity for missense variants

For rare (MAF < 0.01; *n* = 30,065) and very rare (MAF < 0.0001; *n* = 29,498) missense variants across these 34 genes, we applied REVEL and CADD to assess their predicted pathogenicity in the gnomAD dataset (Figure 4 and Supplementary Figure S3). Using REVEL, with a threshold of >0.5 to indicate potential pathogenicity, many genes in the bleeding and fibrinolysis/thrombosis category exhibited median scores below this cutoff (Figure 4A), suggesting that a large proportion of these variants are predicted to have lower deleteriousness according to this metric. Conversely, *MYH9, F11, GGCX, VKORC1,* and *MTHFR* had a median REVEL score higher than the threshold. Analysis of CADD scores yielded relatively similar findings. For both rare and very rare variants, a set of genes, including *GP1BA, F8, FGA, GP6, GP9, F9, F5, F10, SERPINF2,* and *ADAMTS13,* displayed median scores below the commonly used pathogenicity threshold of 20 at the population level (Figure 4C), indicating a lower burden of variants with high predicted deleteriousness based on this metric. In contrast, nearly all genes had a median REVEL and CADD score higher than the thresholds for ClinVar pathogenic/likely pathogenic variants (Figure 4B, D).

**Figure 4:**
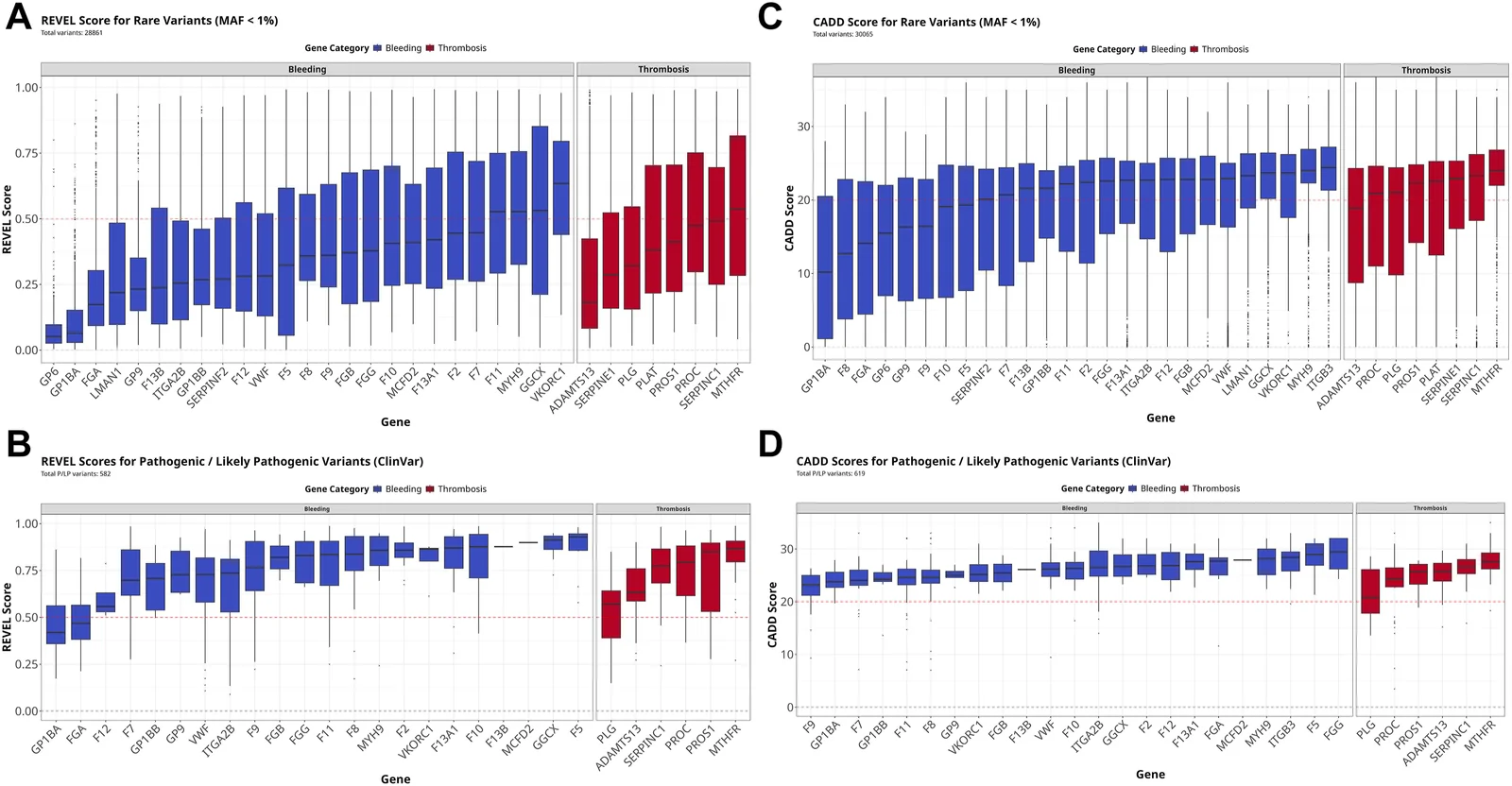
*In silico* prediction of pathogenicity for missense variants in Genome Aggregation Database (gnomAD) and ClinVar. (A) Prediction scores for gnomAD rare (minor allele frequency [MAF] < 0.01) missense variants indicate that most genes have median REVEL scores below the pathogenicity threshold (>0.5), suggesting limited enrichment of highly deleterious variants at the gnomAD population level, whereas a subset of genes (eg, *MYH9, F11, GGCX, VKORC1,* and *MTHFR*) show higher predicted pathogenicity. (B) Conversely, nearly all genes had a median score higher than the threshold for ClinVar pathogenic/likely pathogenic variants. (C) CADD scores demonstrated that most genes (eg, *GP1BA, F8, FGA, GP6, GP9, F9, F5, F10,* and *ADAMTS13*) have median values below the threshold of 20, supporting overall constraint against strongly deleterious missense variation in the general population. (D) In stark contrast, nearly all genes had a median CADD score higher than the threshold for ClinVar pathogenic/likely pathogenic variants.

### 3.5 Evolutionary conservation of rare and very rare variants across coagulation genes

Analysis of phyloP scores for all rare (MAF < 0.01; *n* = 96,300; Figure 5A) and very rare (MAF < 0.0001; *n* = 94,053; Figure S4) variants in the gnomAD general population revealed heterogeneous patterns of evolutionary constraint across hemostasis-related genes. The distributions exhibited clear gene-specific differences. Several genes, such as *GP9*, *LMAN1*, *SERPINF2*, *GGCX*, *GP1BB*, *ITGB3*, *MCFD2, SERPINC1,* and *MTHFR,* showed a relatively small shift toward higher median phyloP values (phyloP > 1), with a greater proportion of variants occurring at evolutionarily conserved sites. In contrast, other genes displayed distributions more tightly centered around neutrality, indicating that most observed population rare variants occur at neutrally evolving or weakly conserved positions (Figure 5A and Supplementary Figure S2). However, none of the 34 genes showed a median ≥2.27. Of note, these patterns should not be interpreted as a direct inverse proxy of gene-level constraint. In highly constrained genes, variants arising at conserved sites are expected to be depleted by purifying selection; however, rare variants represent recent mutational events and may still be observed at constrained positions prior to complete selection. Therefore, the observed phyloP distributions reflect the combined effects of mutational input and ongoing purifying selection.

**Figure 5:**
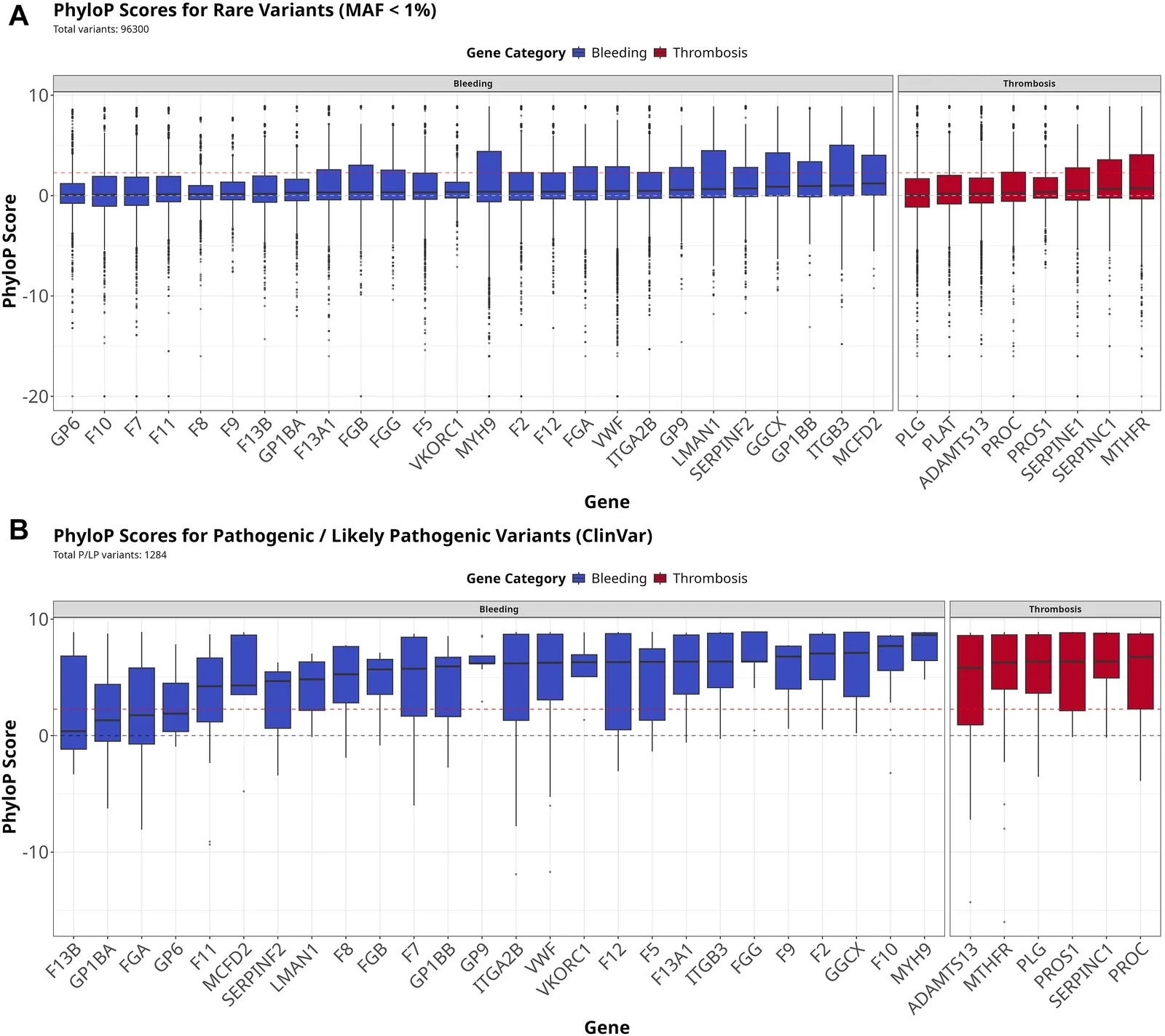
Evolutionary conservation of rare variants across coagulation genes in Genome Aggregation Database and ClinVar. (A) PhyloP score distributions for a total of 91,769 very rare variants in gnomAD reveal gene-specific patterns, with some genes (eg, *GP9, LMAN1, GGCX, GP1BB,* and *SERPINC1*) showing a modest shift toward conserved positions, while most variants occur at neutrally or weakly conserved sites. (B) Inclusion of 1284 ClinVar pathogenic/likely pathogenic variants results in a clear shift toward higher phyloP scores across all genes, indicating enrichment of disease-associated variants at evolutionarily conserved positions and supporting their functional relevance.

Importantly, applying the same analysis to ClinVar pathogenic/likely pathogenic variants identified in gnomAD (*n* = 1284) revealed a pronounced shift toward higher PhyloP scores across the 34 genes, indicating enrichment at evolutionarily conserved sites. This pattern is consistent with the preferential localization of disease-causing variants to functionally constrained positions and further supports their pathogenic relevance (Figure 5B). Overall, while in the gnomAD general population all genes showed little variation at evolutionarily conserved positions—indicating that observed variants predominantly occur in less functionally constrained regions—disease-associated variant data from ClinVar revealed a marked enrichment of these variants within evolutionarily conserved positions across most genes.

### 3.6 Gene-specific heterogeneity in ClinVar variant classifications within population variation

To assess the overlap between ClinVar and gnomAD population variation, we intersected variants reported in ClinVar for these 34 genes with rare and very rare variants identified in the gnomAD dataset. In total, 10,973 ClinVar variants were identified in gnomAD, including pathogenic/likely pathogenic (pathogenic; *n* = 1281), variants of uncertain significance (VUS; *n* = 4578), and benign/likely benign variants (benign; *n* = 4184). Within the 34 genes analyzed, variant classification (pathogenic vs benign) showed a strong dependence on variant type for both rare and very rare variants. For rare variants, LoF variants were almost uniformly pathogenic, except for *PLAT*. Notably, no ClinVar LoF variants were observed in gnomAD for *VKORC1* and *SERPINE1*. In contrast and as expected, synonymous variants were mainly benign, with only a few exceptions in *F8*, *F9*, *F5*, *VWF*, and *MTHFR* (Figure 6A). Missense variants, however, displayed marked gene-specific heterogeneity, with varying proportions of benign and pathogenic classifications. For example, the majority of missense variants in *F10*, *F11*, *F2*, *F7*, *F8*, *ITGA2B*, *ITGB3*, *MCFD2*, *VWF*, *MTHFR*, *PROC*, *PROS1*, and *SERPINC1* were classified as pathogenic. In contrast, most to nearly all missense variants in *F12*, *F13B*, *F5*, *FGB*, *GP1BA*, *GP6*, *LMAN1*, *MYH9*, *SRPINF2, PLAT,* and *SERPINE1* were benign, underscoring substantial differences in tolerance to amino acid variation across genes (Figure 6A). Indeed, across all 34 genes, benign variation was mainly composed of synonymous variants, with few exceptions where missense variants (*LMAN1* and *VKORC1*) predominated. In contrast, pathogenic variants exhibited a gene-specific distribution of both LoF and missense classes, indicating distinct mutational mechanisms underlying related diseases across genes (Figure 6B). The same analysis restricted to very rare variants (MAF < 0.0001) is shown in Supplementary Figure S5.

**Figure 6:**
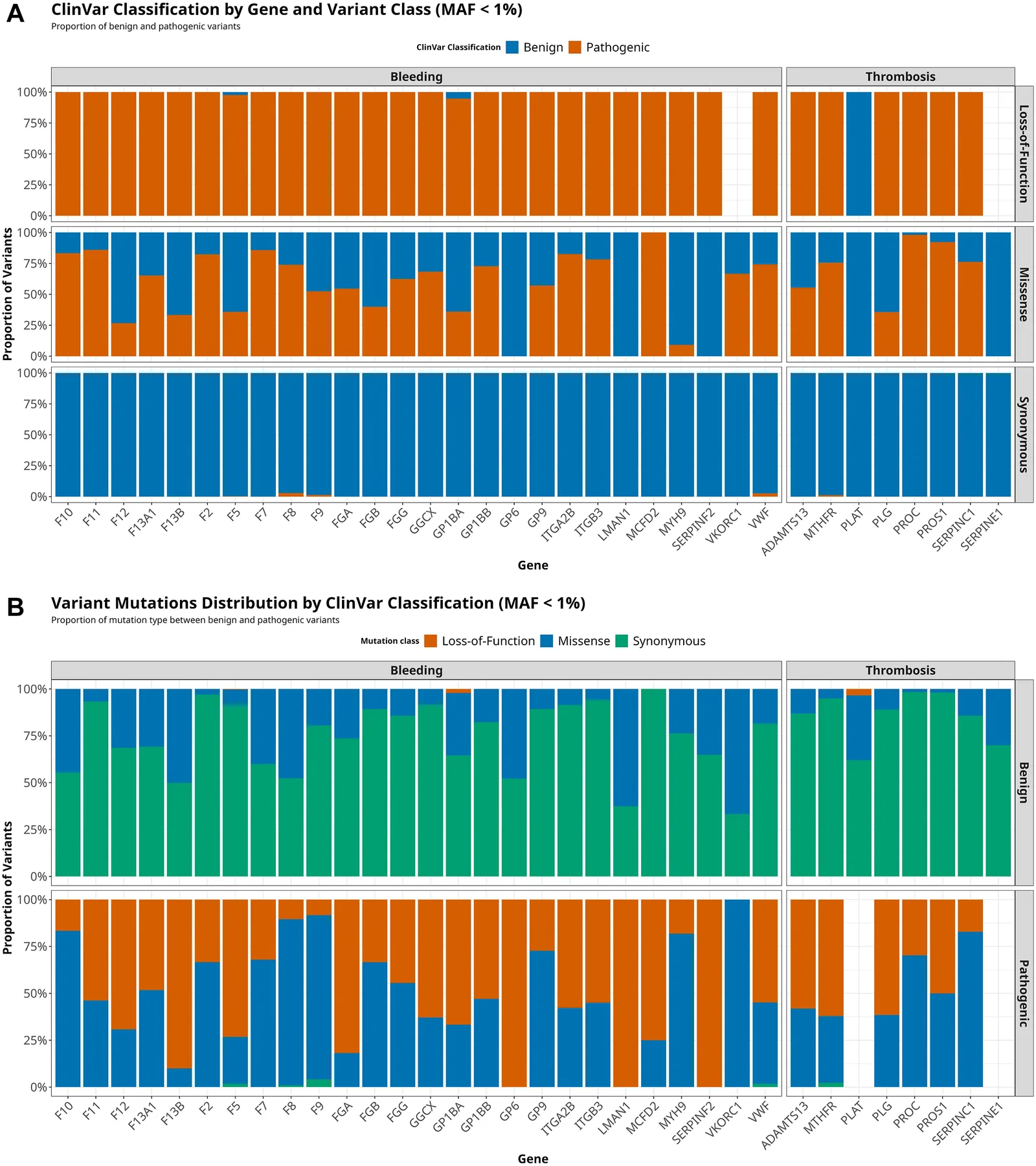
Gene-specific heterogeneity in ClinVar variant classifications within population variation. (A) Intersection of ClinVar and Genome Aggregation Database rare variants reveals strong dependence of classification on variant type, with loss-of-function variants predominantly pathogenic and synonymous variants largely benign across genes. Missense variants show marked gene-specific heterogeneity, with some genes (eg, *F8, F9, F2, F10,* and *VWF*) enriched for pathogenic classifications, while others (eg, *F12, F5, GP6,* and *LMAN1*) are predominantly benign. (B) Overall, benign variation is largely driven by synonymous variants, whereas pathogenic variants reflect gene-specific contributions of both loss-of-function and missense mechanisms. MAF, minor allele frequency.

We next quantified the proportion of pathogenic vs benign rare variants (MAF < 0.01) annotated in ClinVar across genes represented in gnomAD (Figure 7A). Among bleeding-associated genes, benign variants predominated in most cases; however, several genes, including *MCFD2, F7, F10, F13B, FGA,* and *F8*, exhibited a substantially higher proportion of pathogenic variants, exceeding 60%. In thrombosis-associated genes, benign variants similarly dominated overall, although *PROC* showed a relative enrichment of pathogenic variants (∼55%). Conversely, a subset of genes, including *MYH9, SERPINF2, FGB, PLAT,* and *SERPINE1*, harbored ≤10% pathogenic ClinVar variants in gnomAD. Collectively, these findings highlight pronounced gene-level heterogeneity in the burden of pathogenic variation, consistent with differences in selective constraint, disease mechanisms, and tolerance to deleterious variants in the general population. The same analysis restricted to very rare variants (MAF < 0.0001) is shown in Supplementary Figure S6.

**Figure 7:**
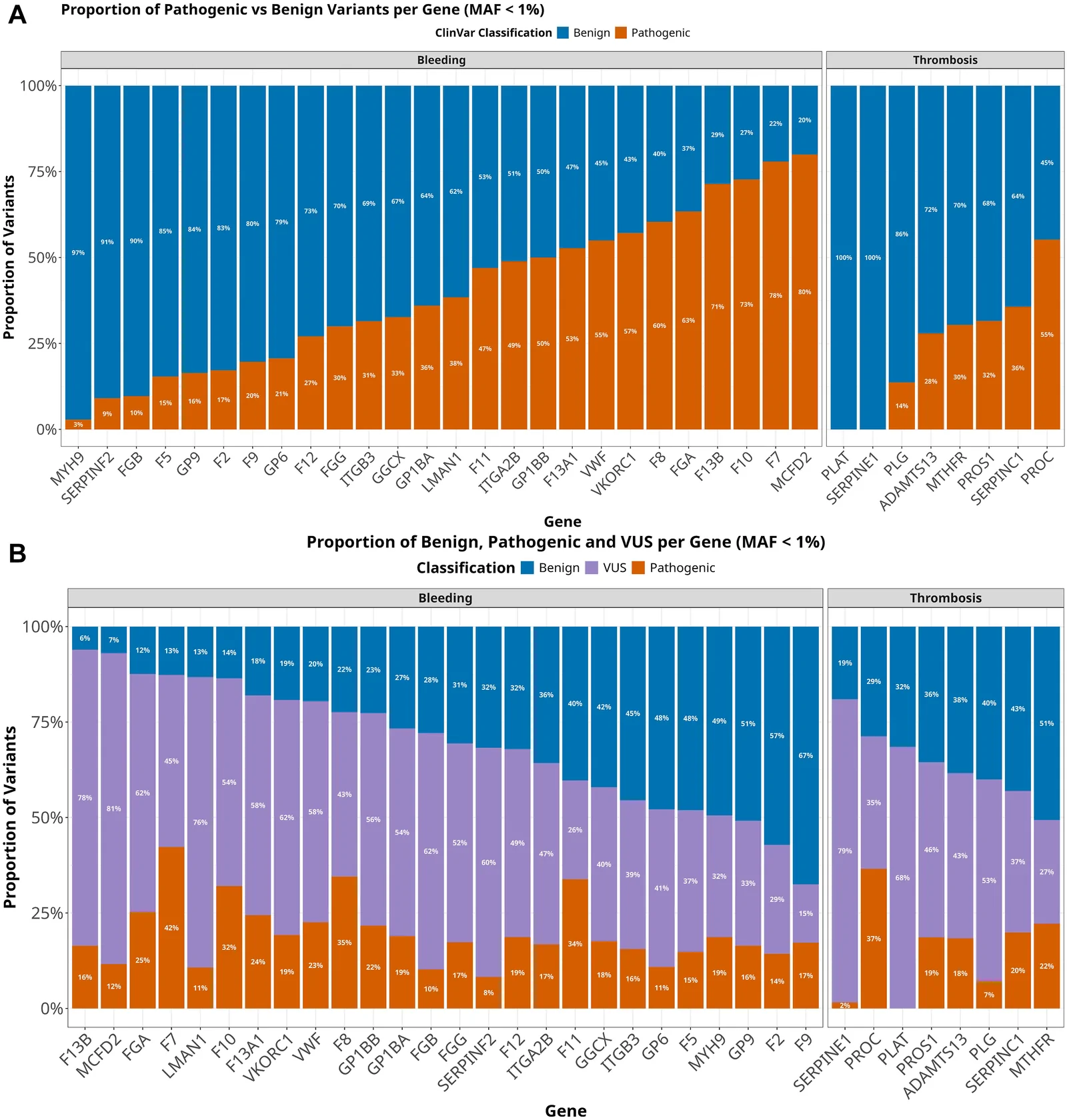
Proportion of pathogenic vs benign rare variants across coagulation genes in Genome Aggregation Database. (A) Analysis of ClinVar-annotated rare variants (minor allele frequency [MAF] < 0.01) in Genome Aggregation Database reveals substantial gene-level heterogeneity, with most bleeding- and thrombosis-associated genes dominated by benign variants, while others (eg, *F7, F10, F13B, FGA, F8,* and *MCFD2*) show enrichment of pathogenic variants. (B) Incorporating predicted deleterious variants (CADD > 20 and REVEL > 0.5) further highlights distinct gene-specific patterns, with some genes (eg, *F7, F8, F11,* and *PROC*) enriched for pathogenic or high-impact variants, whereas others (eg, *F9, F2, GP9, MYH9,* and *F5*) are predominantly benign, underscoring differences in tolerance to variation and disease burden across genes. VUS, variants of uncertain significance.

Investigating ClinVar rare and very rare variants in gnomAD, including pathogenic, VUS with deleterious *in silico* predictions (CADD > 20 and REVEL > 0.5), and benign, the coagulation genes show wide variation in the proportion of pathogenic vs benign variants (Figure 7B and Supplementary Figure S4). Most genes showed a predominance of VUS, while the proportion of pathogenic variants varied considerably, ranging from genes enriched for pathogenic classifications (eg, *F7, F10, F11, F8, FGA,* and *PROC*) to genes with a higher proportion of benign variants (eg, *F9, F2, GP6, MTHFR,* and *SERPINC1*). These findings highlight substantial heterogeneity in the clinical annotation landscape of hemostasis-related genes.

Finally, we examined the combined burden of ClinVar pathogenic variants and VUS with deleterious *in silico* predictions among both rare (Figure 8) and very rare variants (Supplementary Figure S7) across genes. Pathogenic variants predominated in most genes, although the VUS with deleterious *in silico* predictions group contributed substantially in several genes, particularly *PLAT*, *SERPINE1*, *FGB*, *MCFD2*, and *LMAN1*. When normalized by CDS length (right panel), the number of pathogenic variants per CDS length varied markedly across genes, with some (eg, *PROC*, *MTHFR*, *F11, F7,* and *MYH9*) showing a high burden of pathogenic and predicted pathogenic variation in the general population, whereas others (eg, *SERPINF2*, *SERPINE1*, *LMAN1, PLAT, GP1BA, GP6*, and *F13B*) showed a relatively low burden. These results highlight pronounced gene-level differences in both variant interpretation and putative pathogenic variant burden in the general population.

**Figure 8:**
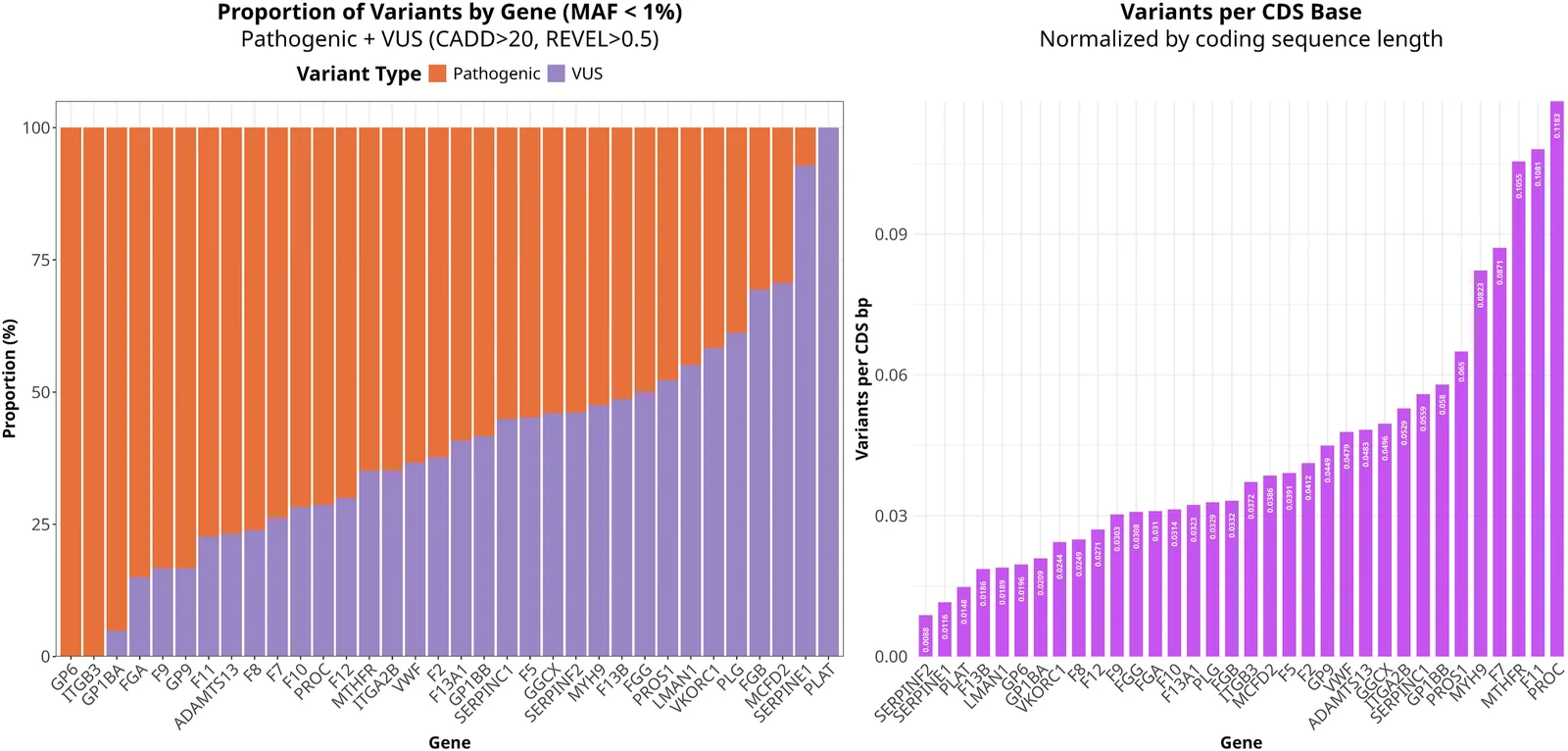
Combined burden of ClinVar pathogenic and predicted pathogenic variants of uncertain significance (VUS) variants across coagulation genes in Genome Aggregation Database. Pathogenic variants dominate in most genes, with predicted pathogenic VUS contributing substantially in select genes, including *SERPINE1, FGB, MCFD2,* and *LMAN1*. Normalization by coding sequence (CDS) length of each gene reveals marked gene-specific differences in variant density, with genes such as *PROC, MTHFR, F11, F7,* and *MYH9* carrying a high burden of pathogenic and predicted pathogenic variants, whereas others (eg, *SERPINF2*, *SERPINE1,PLAT,LMAN1, GP1BA, GP6,* and *F13B*) show relatively low density. These findings underscore gene-level heterogeneity in both variant interpretation and putative pathogenic variant burden in the general population. MAF, minor allele frequency.

## 4 Discussion

Our study reveals that genes involved in hemostasis exhibit a continuous spectrum of evolutionary constraint and tolerance to genetic variation, challenging the implicit assumption that the coagulation system is uniformly constrained. By integrating population-scale variation from more than 800,000 individuals with constraint metrics, *in silico* pathogenicity prediction, evolutionary conservation, and clinical annotation across 34 established hemostatic genes, we provide a unified framework for understanding the genetic architecture of coagulation, platelet function, fibrinolysis, and thrombosis.

A key finding is that hemostatic genes do not segregate into discrete categories but instead exhibit a continuum of selective constraint. At one end of this spectrum are highly constrained core components, including *F8, F9, F2, VWF, FGG, FGB, F5, SERPINC1, PROS1*, and *MYH9*, which show strong depletion of LoF and missense variation and strong signals of evolutionary conservation. At the opposite end are genes such as *GP6, GP1BB, F12, F11, VKORC1, MCFD2*, and *LMAN1*, which display relative tolerance to variation across multiple metrics. Importantly, this stratification is not metric-specific but is consistently observed across independent analytical frameworks, indicating that it reflects a robust biological feature of the hemostatic system. These differences likely reflect variation in the functional importance and clinical consequences of disruption among individual components of the hemostatic pathway. For example, highly constrained genes such as *F2, F5, F8, F9, VWF, SERPINC1*, and *PROS1* are associated with severe bleeding or thrombotic phenotypes when disrupted, whereas genes such as *F11* and *F12*, which exhibited greater tolerance to variation, are often associated with milder or even asymptomatic phenotypes. Similarly, *GP6*, a receptor involved in platelet activation, may tolerate greater genetic variation because alternative platelet activation pathways can partially compensate for its loss. This heterogeneity is consistent with comparative genomic evidence showing that hemostasis genes are not uniformly conserved across species. For example, birds possess fewer hemostatic genes than mammals, particularly among platelet-related pathways, suggesting that evolutionary importance varies across components of the hemostatic system [18].

This continuum of constraint is further supported by rare variant burden analyses, which demonstrate substantial gene-specific differences even after normalization for CDS length. Genes under strong selective constraint show reduced variant density, whereas more tolerant genes accumulate a higher burden of rare variation in the gnomAD population. Importantly, *in silico* pathogenicity prediction further supports this framework, as highly constrained genes are depleted for variants with high predicted deleteriousness, consistent with stronger purifying selection against damaging alleles. Our finding that constrained hemostatic genes have reduced rare variant density aligns with observations across disease gene sets, demonstrating that gene-level constraint metrics broadly identify depletion of damaging variants and prioritize Mendelian disease genes across pathways [19,20].

Evolutionary conservation analyses provide an additional and complementary layer of evidence. While most rare variants in the general population occur at neutrally evolving or weakly constrained sites, gene-specific enrichment patterns are observed, reflecting the interaction between mutational input and purifying selection. Notably, clinically classified pathogenic variants from ClinVar are significantly shifted toward evolutionarily conserved positions across all genes, reinforcing the biological relevance of constraint signals and distinguishing disease-associated variation from background population diversity. The concordance between population variation, evolutionary conservation, and clinical annotations further supports the validity of the observed constraint spectrum [21].

From an evolutionary perspective, these findings support a model in which the hemostatic system is shaped by opposing selective pressures, where both bleeding and thrombosis confer fitness disadvantages. This evolutionary tension likely contributes to the observed heterogeneity in constraint, with some genes evolving under strong purifying selection due to essential roles in hemostasis, while others retain greater flexibility due to redundancy or modulatory functions within the pathway. This evolutionary framework provides a mechanistic explanation for the constraint continuum observed across genes.

Our results further highlight that ClinVar annotations themselves reflect strong gene-specific heterogeneity. While LoF variants are almost uniformly classified as pathogenic, missense variants show substantial variability in clinical interpretation across genes, and synonymous variants are predominantly benign. This suggests that pathogenicity is not only variant-class dependent but also strongly influenced by gene context, molecular mechanism, and tolerance to functional perturbation. Moreover, the presence of pathogenic annotations in population databases underscores that clinical classification does not always map directly onto population-level constraint or disease penetrance, reinforcing the need for gene-specific interpretation frameworks. The marked differences in the relative proportions of pathogenic, benign, and VUS classifications across genes further suggest that the clinical annotation landscape is shaped by gene-specific disease mechanisms, penetrance, and historical patterns of variant interpretation.

Beyond providing insights into the evolutionary genetics of hemostasis, these findings may also have relevance for variant interpretation. Genes exhibiting strong depletion of missense or LoF variation are expected to be less tolerant of novel rare variants, whereas variants identified in more tolerant genes may require additional evidence for confident pathogenicity assessment. Although constraint metrics should not be used in isolation, they provide valuable context for the interpretation of genetic variation in inherited bleeding and thrombotic disorders. Our proposal to integrate constraint metrics into variant interpretation aligns with recent pathogenicity predictors that explicitly model regional and gene-level constraint [22,23].

This study has several limitations. All analyses were performed using gnomAD v4.1.1 constraint metrics, except for *GP9* LoF variants, for which data from gnomAD v2.1.1 were used due to the unavailability of corresponding data in gnomAD v4.1.1. Ancestry-stratified analyses were not performed, which may mask population-specific effects. gnomAD does not include phenotypic information, limiting clinical interpretation of variants. ClinVar annotations and *in silico* prediction tools have variable reliability and incomplete coverage; therefore, results should be interpreted with caution. Sex-specific analyses of X-linked genes were limited by incomplete sex-stratified data. Of note, comparisons of variant burden across genes should be interpreted cautiously. Although variant counts were normalized by CDS length, residual differences may reflect factors not accounted for in the analysis, including variation in sequence coverage, callable coding regions, local mutation rates, and gene architecture. Finally, because per-base coverage and callable-region data were not available in the aggregated gnomAD dataset used for this study, it was not possible to adjust variant counts for gene-specific differences in sequencing accessibility.

In conclusion, our new findings demonstrate that genes involved in hemostasis exhibit substantial heterogeneity in selective constraint, evolutionary conservation, and tolerance to genetic variation. By integrating population-scale genomic data, gene-level constraint metrics, evolutionary conservation, and clinical variant annotations across 34 established hemostatic genes, this study provides a comprehensive overview of the genetic architecture underlying bleeding, thrombotic, and fibrinolytic disorders. These results highlight that different components of the hemostatic system are subject to distinct evolutionary pressures and mutational constraints, reflecting their diverse biological functions and disease mechanisms. Collectively, these findings establish a gene-specific framework for understanding variation tolerance across the hemostatic system and provide an evolutionary context for the interpretation of rare variants in inherited bleeding and thrombotic disorders.
