# Supplementary material for: Evolutionary constraint and population variation across coagulation genes
Source: Res Pract Thromb Haemost. 2026 Sep 24;10(7):106980. doi: 10.1016/j.rpth.2026.106980 (PMC13696369; doi:10.1016/j.rpth.2026.106980)
Supplement: Supplementary file 1 [file mmc1.pdf]

Supplementary files

Evolutionary constraint and population variation across coagulation genes

Omid Seidizadeh[1], Himani Chand[1], Andrea Cairo[1], Flora Peyvandi[1,2]

1. Fondazione IRCCS Ca'Granda Ospedale Maggiore Policlinico, Angelo Bianchi Bonomi Hemophilia and Thrombosis Center, Milan, Italy
2. Department of Pathophysiology and Transplantation, Università degli Studi di Milano, Milan, Italy

Running title: Evolutionary constraint of coagulation genes

Correspondence

Omid Seidizadeh, PhD

Fondazione IRCCS Ca'Granda Ospedale Maggiore Policlinico, Angelo Bianchi Bonomi Hemophilia and Thrombosis Center, Department of Pathophysiology and Transplantation, Università degli Studi di Milano, Via Pace 9, 20122 Milan, Italy.

Email: omidseidi93@gmail.com, omid.seidizadeh@unimi.it, phone: +390250320288

Table S1. Genes included in the study, grouped by function, with encoded proteins and associated disorders

Category	Gene	Transcript	Protein	Associated disease(s)	Inheritance
**Coagulation factors**	*F2*	ENST00000311907	Prothrombin	Prothrombin deficiency; thrombophilia	AR
	F5	ENST00000367797	Factor V	Factor V deficiency; Factor V Leiden thrombophilia	AR
	F7	ENST00000375581	Factor VII	Factor VII deficiency	AR
	F8	ENST00000360256	Factor VIII	Hemophilia A	X-linked
	F9	ENST00000218099	Factor IX	Hemophilia B	X-linked
	F10	ENST00000375559	Factor X	Factor X deficiency	AR
	F11	ENST00000403665	Factor XI	Factor XI deficiency	AR
	F12	ENST00000253496	Factor XII	Factor XII deficiency	AR
	F13A1	ENST00000264870	Factor XIII A subunit	Factor XIII deficiency	AR
	F13B	ENST00000367412	Factor XIII B subunit	Factor XIII deficiency	AR
**Fibrinogen complex**	*FGA*	ENST00000403106	Fibrinogen α chain	Congenital fibrinogen disorders	AR / AD
	FGB	ENST00000302068	Fibrinogen β chain	Congenital fibrinogen disorders	AR / AD
	FGG	ENST00000336098	Fibrinogen γ chain	Congenital fibrinogen disorders	AR / AD
**Vitamin K pathway**	*GGCX*	ENST00000233838	Gamma-glutamyl carboxylase	Combined vitamin K–dependent factor deficiency	AR
	VKORC1	ENST00000394975	Vitamin K epoxide reductase complex subunit 1	Vitamin K–dependent factor deficiency; warfarin resistance	AR
**Platelet receptors and function**	*GP1BA*	ENST00000329125	Platelet glycoprotein Ibα	Bernard–Soulier syndrome<br>PT-VWD	AR<br>AD
	GP1BB	ENST00000366425	Platelet glycoprotein Ibβ	Bernard–Soulier syndrome	AR
	GP9	ENST00000307395	Platelet glycoprotein IX	Bernard–Soulier syndrome	AR
	GP6	ENST00000417454	Platelet glycoprotein VI	Platelet-type bleeding disorder	AR / AD
	ITGA2B	ENST00000262407	Integrin αIIb	Glanzmann thrombasthenia	AR
	ITGB3	ENST00000559488	Integrin β3	Glanzmann thrombasthenia	AR
	MYH9	ENST00000216181	Non-muscle myosin heavy chain IIA	MYH9-related disorders	AD
**von Willebrand factor**	*VWF*	ENST00000261405	von Willebrand factor	von Willebrand disease	AD / AR
**Protein trafficking**	*LMAN1*	ENST00000251047	ERGIC-53	Combined FV and FVIII deficiency	AR

**(FV/FVIII secretion)**	*MCFD2*	ENST00000319466	MCFD2 protein	Combined FV and FVIII deficiency	AR
**Fibrinolysis**	*PLG*	ENST00000308192	Plasminogen	Plasminogen deficiency	AR
	PLAT	ENST00000220809	Tissue plasminogen activator	Fibrinolytic disorders	AD (rare)
	SERPINF2	ENST00000324015	Alpha-2 antiplasmin	Alpha-2 antiplasmin deficiency	AR
	SERPINE1	ENST00000223095	Plasminogen activator inhibitor-1	PAI-1 deficiency	AR
**Thrombosis and anticoagulation**	*PROC*	ENST00000234071	Protein C	Protein C deficiency	AD / AR
	PROS1	ENST00000394236	Protein S	Protein S deficiency	AD
	SERPINC1	ENST00000367698	Antithrombin	Antithrombin deficiency	AD
	ADAMTS13	ENST00000371929	ADAMTS13 metalloprotease	Thrombotic thrombocytopenic purpura	AR
	MTHFR	ENST00000376590	Methylenetetrahydrofolate reductase	Hyperhomocysteinemia; thrombosis risk	AR

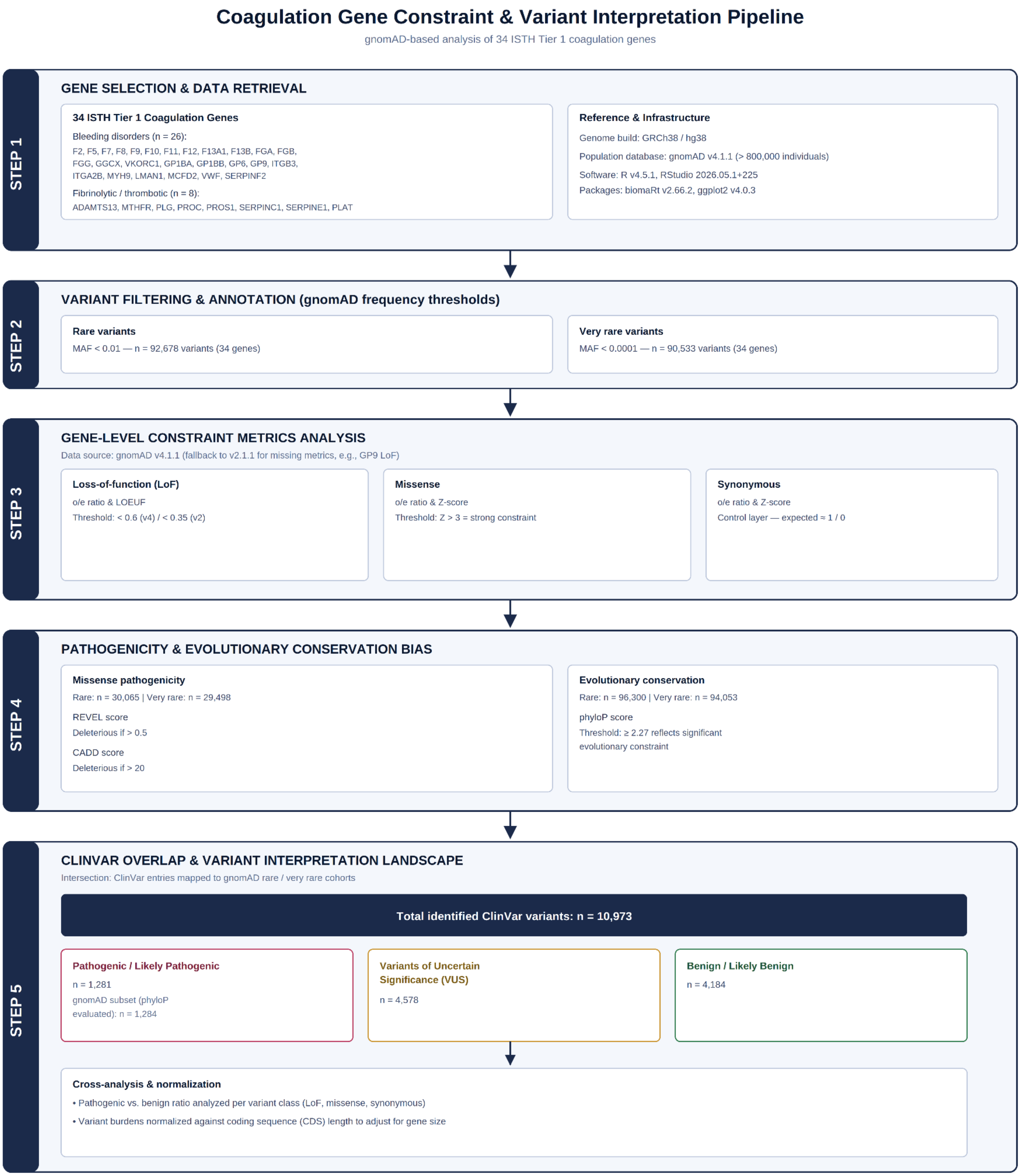

Figure S1. Flowchart of data analysis process.

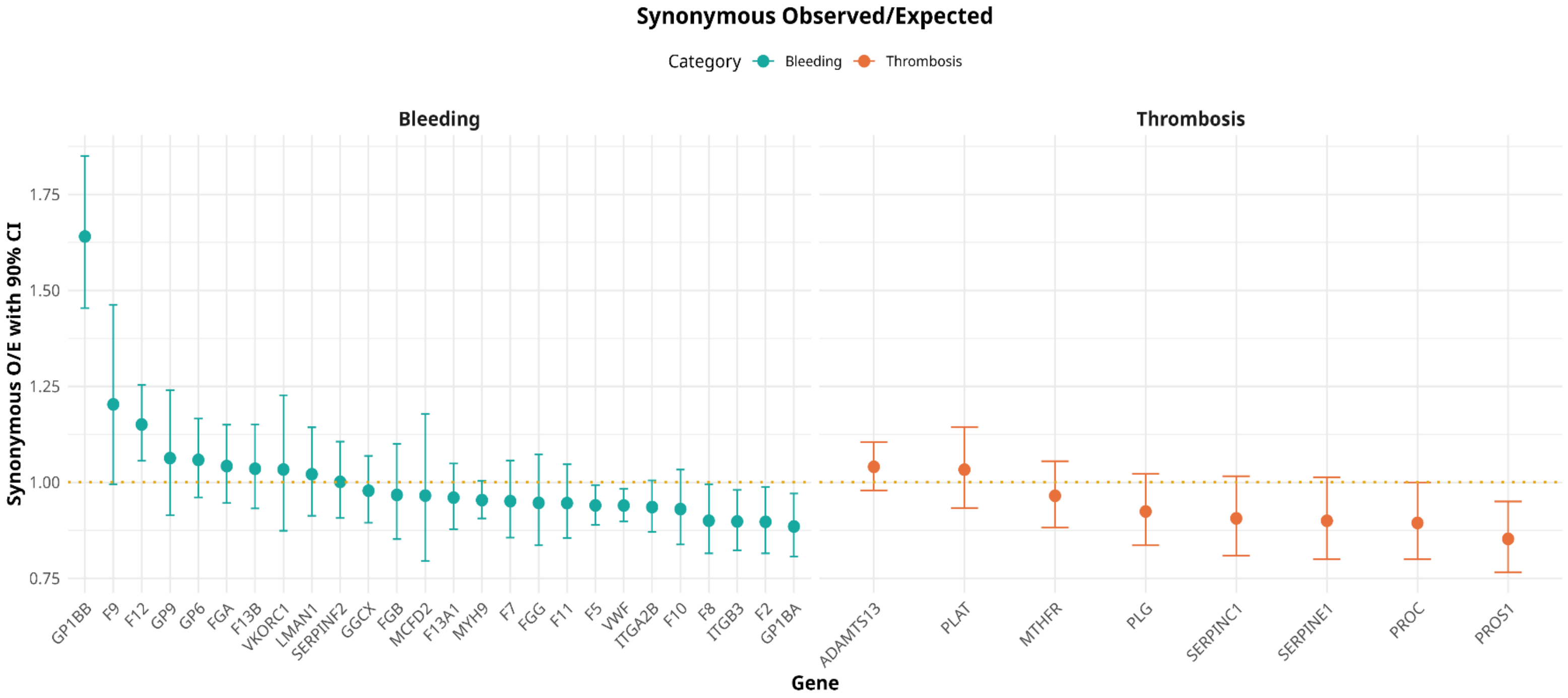

Figure S2. Observed/expected ratio for synonymous variants across the genes.

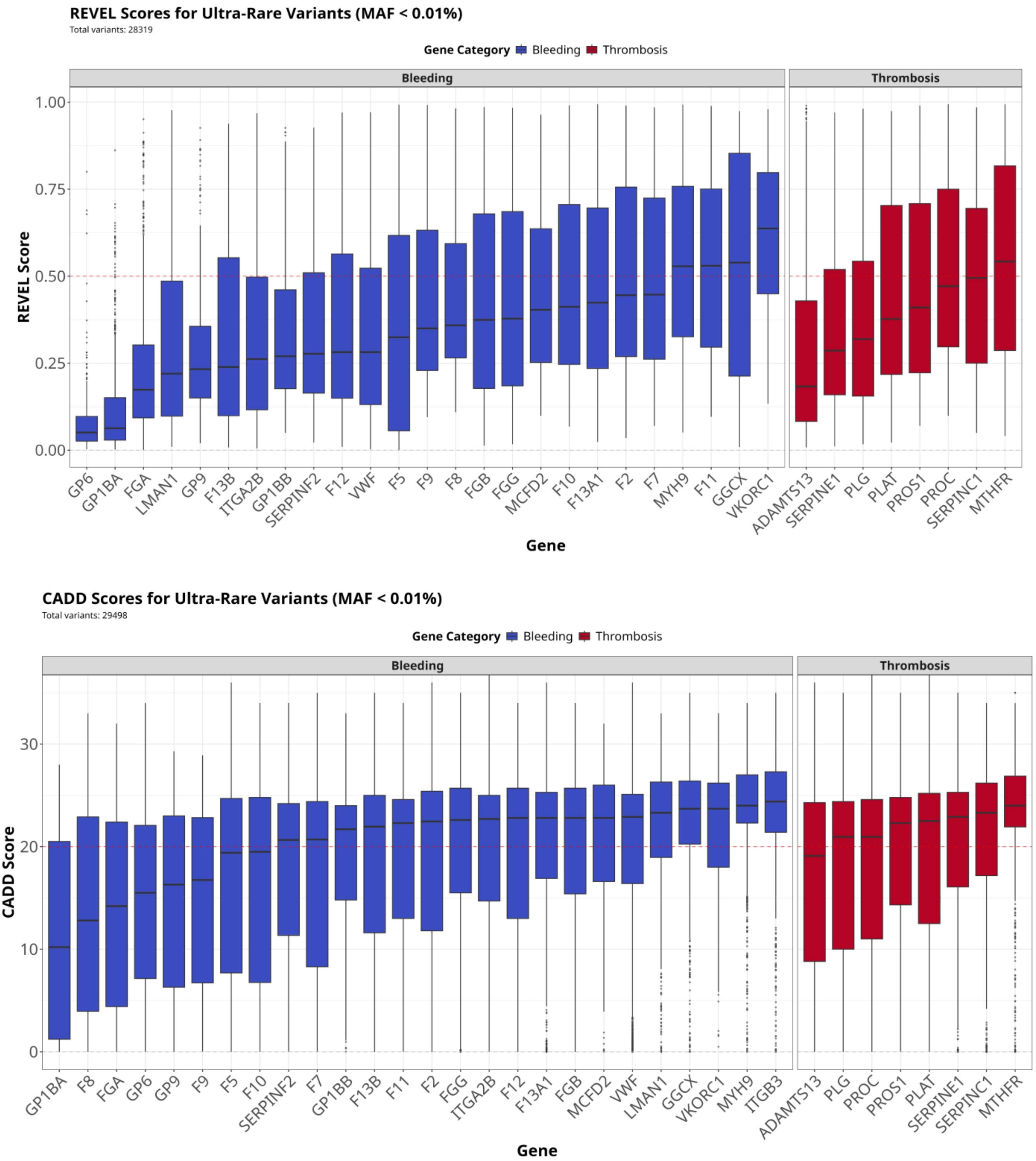

Figure S3. In silico prediction of pathogenicity for very rare missense variants in gnomAD.

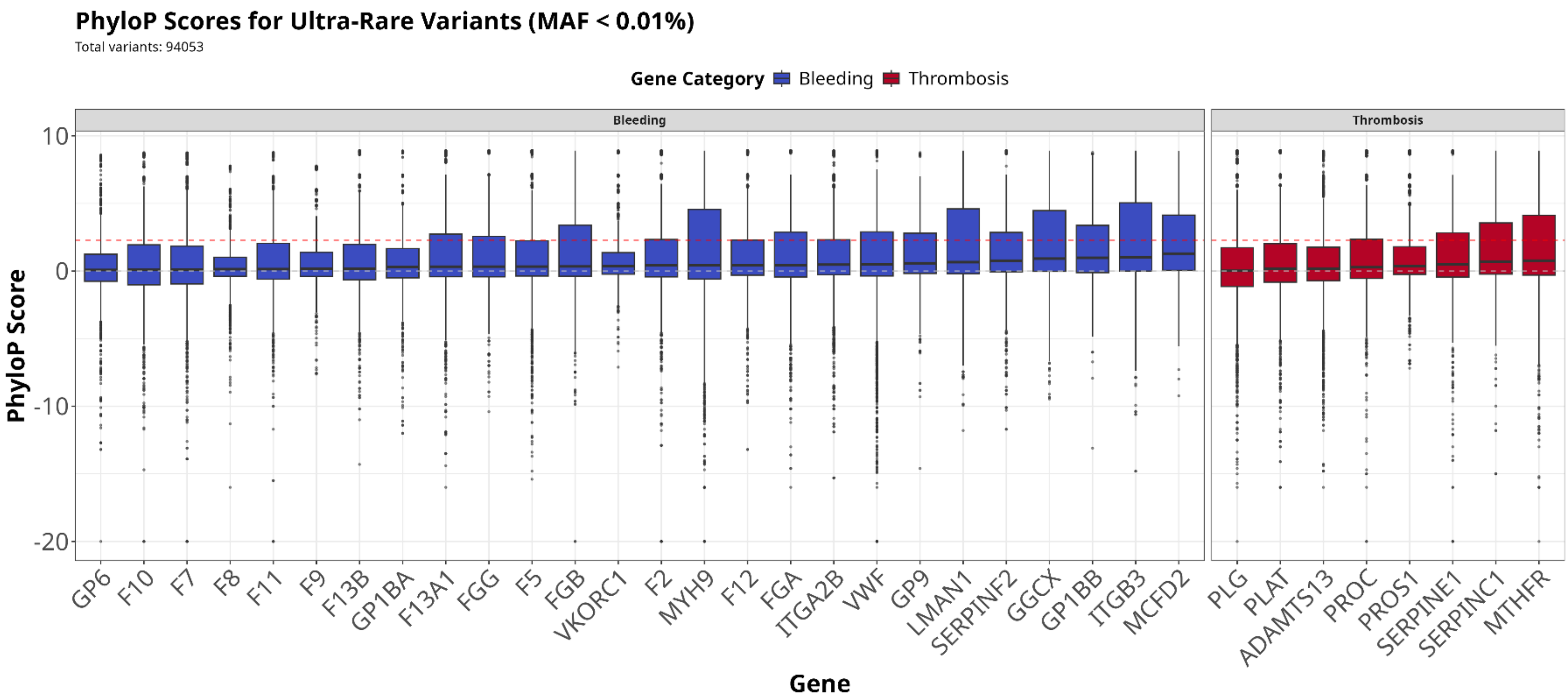

Figure S4. Evolutionary conservation of very rare variants across coagulation genes.

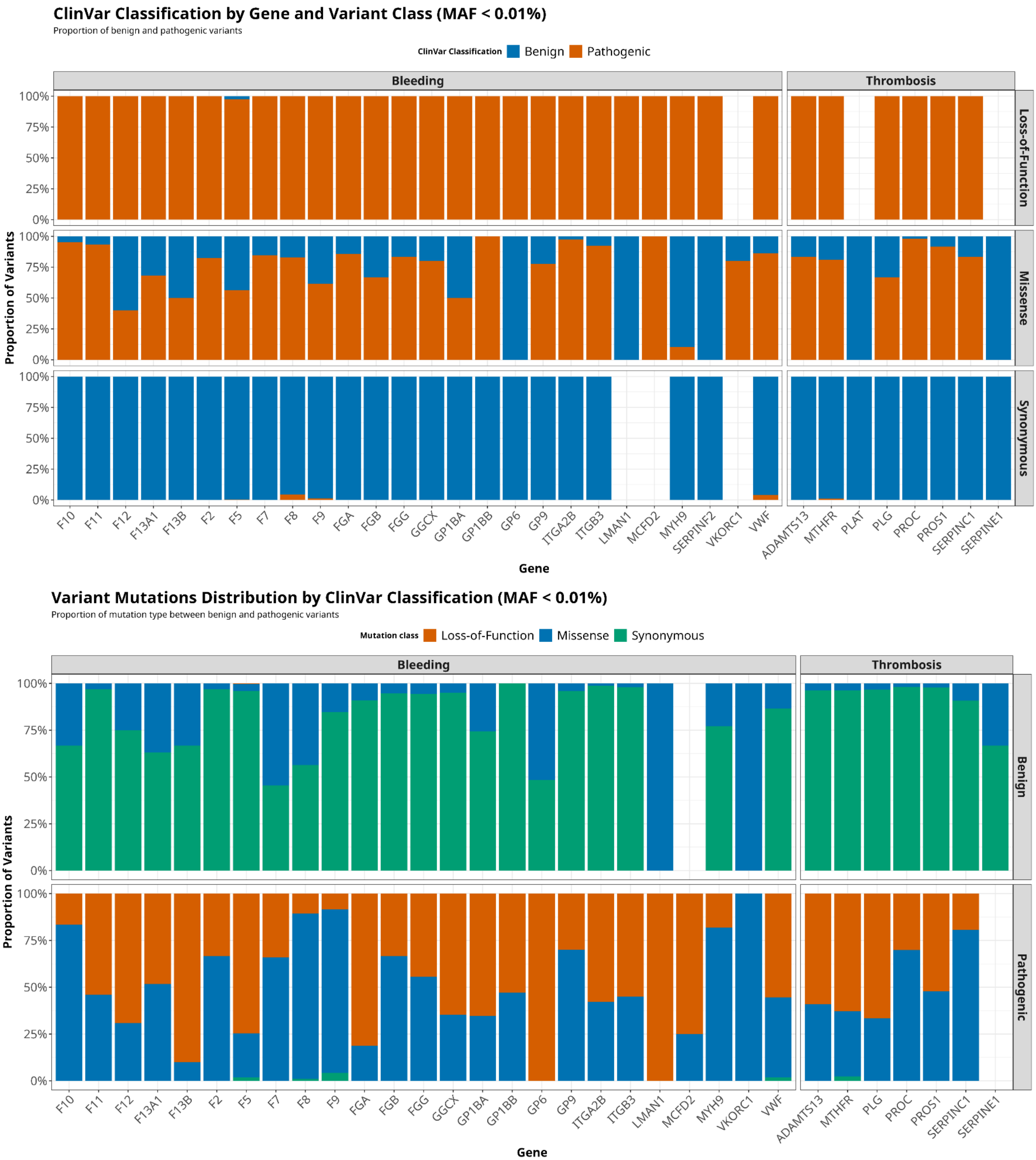

Figure S5. Gene-specific heterogeneity in very rare ClinVar variant classifications within population variation.

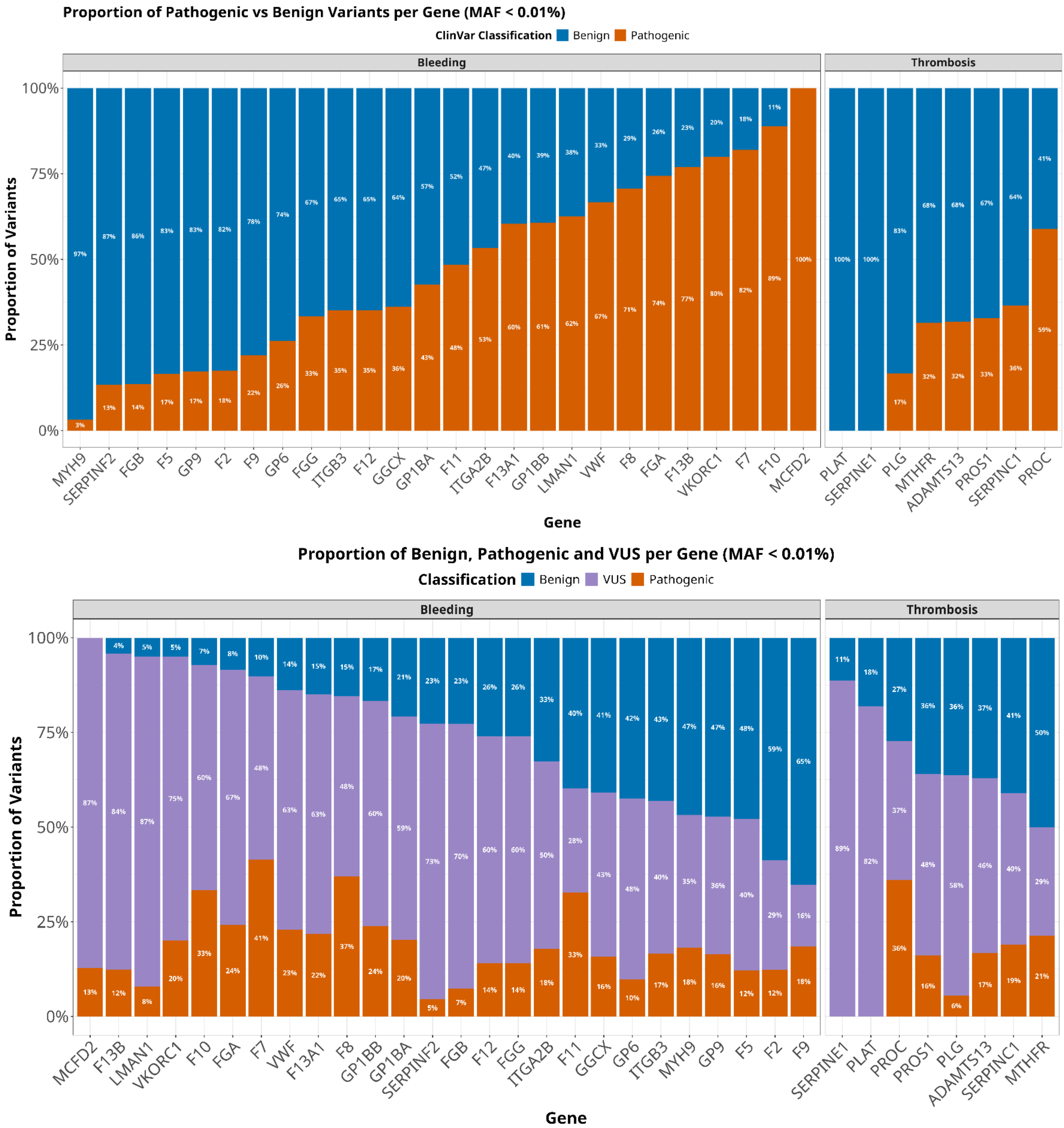

Figure S6. Proportion of pathogenic versus benign very rare variants across coagulation genes.

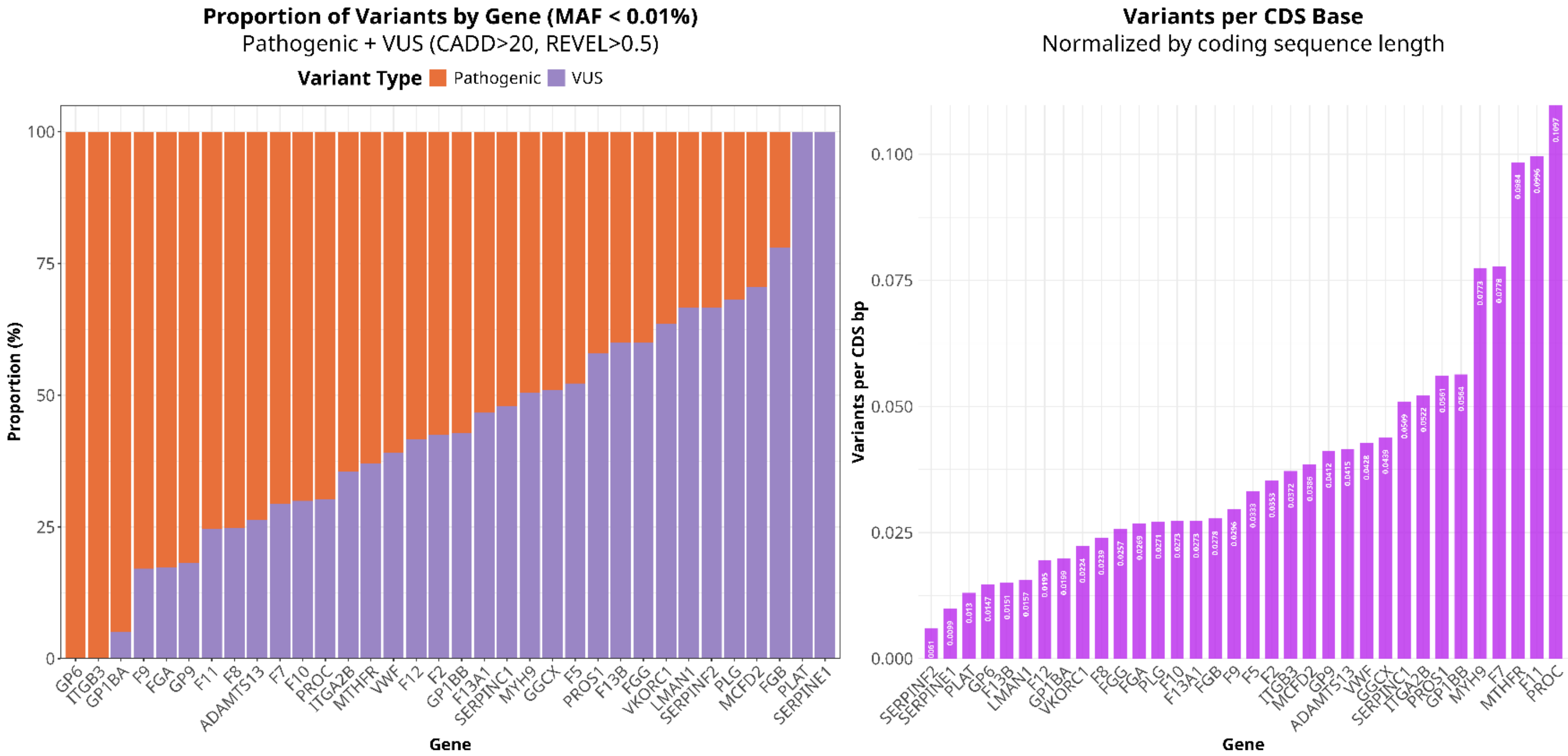

Figure S7. Combined burden of very rare ClinVar pathogenic and predicted pathogenic VUS variants across coagulation genes.
